# Comprehensive Characterization of Shredded Lithium‐Ion Battery Recycling Material

**DOI:** 10.1002/chem.202200485

**Published:** 2022-03-14

**Authors:** Christoph Peschel, Stefan van Wickeren, Yves Preibisch, Verena Naber, Denis Werner, Lars Frankenstein, Fabian Horsthemke, Urs Peuker, Martin Winter, Sascha Nowak

**Affiliations:** ^1^ University of Münster MEET Battery Research Center Corrensstraße 46 48149 Münster Germany; ^2^ TU Bergakademie Freiberg Institute of Mechanical Process Engineering and Mineral Processing Agricolastraße 1 09599 Freiberg Germany; ^3^ Helmholtz-Institute Münster, IEK-12 Forschungszentrum Jülich Corrensstraße 46 48149 Münster Germany

**Keywords:** elemental analysis, lithium-ion battery, recycling, recycling material characterization, reverse engineering, speciation

## Abstract

Herein we report on an analytical study of dry‐shredded lithium‐ion battery (LIB) materials with unknown composition. Samples from an industrial recycling process were analyzed concerning the elemental composition and (organic) compound speciation. Deep understanding of the base material for LIB recycling was obtained by identification and analysis of transition metal stoichiometry, current collector metals, base electrolyte and electrolyte additive residues, aging marker molecules and polymer binder fingerprints. For reversed engineering purposes, the main electrode and electrolyte chemistries were traced back to pristine materials. Furthermore, possible lifetime application and accompanied aging was evaluated based on target analysis on characteristic molecules described in literature. With this, the reported analytics provided precious information for value estimation of the undefined spent batteries and enabled tailored recycling process deliberations. The comprehensive feedstock characterization shown in this work paves the way for targeted process control in LIB recycling processes.

## Graphical abstract

**Comprehensive characterization** of shredded lithium‐ion battery (LIB) recycling material was performed. As a result, a toolbox for analysis of these complex samples compromising various electrolyte, active and inactive materials is provided. By means of extraction and pyrolysis with subsequent chromatography and mass spectrometry‐based analyses for example identification of hazardous species and polymer binder residues on trace level was enabled. Thus, detailed feedstock characterization serves as a starting point for risk assessment, tailored material treatment and targeted process control within LIB recycling. Comprehensive Characterization of Shredded Lithium‐Ion Battery Recycling Material (Nowak et al.) #ProjektDEAL

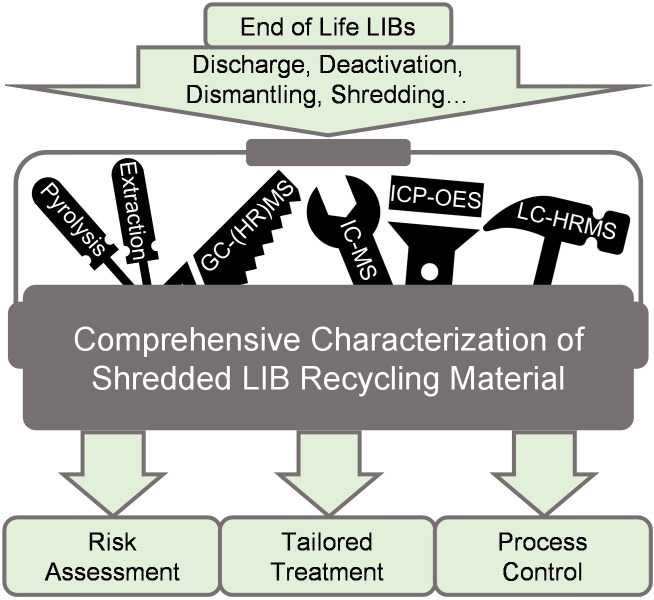

## Introduction

Since its commercialization three decades ago, the lithium‐ion battery (LIB) has been a key technology to achieve a digitalized 21^st^ century. Hand‐held consumer electronics are battery powered examples for this. Improvements in energy and power density, cycle and calendar life, energy efficiency and safety also shifted application of LIBs towards electromobility to achieve greener mobility.[^1^, ^2^, ^3^] After first restraint, (plug‐in) hybrid electric vehicles ((P)HEVs) and fully battery powered electric vehicles (BEVs) are gaining popularity. Reasons are manifold, ranging from an increase of social ecological awareness over improved LIB performance to lowered acquisition costs, partially enabled by government subsidies.^[2]^

Starting mainly from ecological motivations enforced by law, original equipment manufacturers (OEMs) also realized the economic potential of electromobility and expand their EV portfolios.^[4]^ Beyond that, OEMs also invest in battery cell production to meet their rising demands.[^2^, ^5^, ^6^] Accompanied with the massive growth of LIB cell production and application, the amounts of end‐of‐life LIBs will also increase time‐shifted. Therefore, recycling of LIBs will play an important role, not only to further reduce the ecological footprint of electromobility, but also for a more secured raw material supply of geographically unevenly distributed elements like cobalt and nickel.[^7^, ^8^, ^9^, ^10^, ^11^] However, to lower the overall ecological footprint of LIBs, also recycling process need to be improved or even regulated regarding sustainability for example by moving towards circular processes.[^12^, ^13^]

One current state‐of‐the‐art LIB recycling procedure can start with deactivation and shredding of the spent battery modules. After discharge and dismantling, modules are shredded under inert conditions to avoid thermal runaways and volatile electrolyte residues are removed.[^14^, ^15^, ^16^, ^17^, ^18^] Afterward, hydrometallurgical procedures and classification are applied to regain valuable active and inactive materials. If the recycling process starts with pyrometallurgical treatment, discharge and deactivation are not mandatory, but are in some cases performed.[^16^, ^19^, ^20^, ^21^] Detailed organization and implementation of future LIB recycling on industrial scales is not clear, yet. In addition, responsibilities for complying with the required recycling rates remain unclear. Presumably, decentralized deactivation and larger recycling plants will combine safe treatment and transport of spent LIBs with the use of economy of scale for recycling plants.[^10^, ^22^]

LIB material characterization is inevitable in the context of material recycling. Evaluation and adjustment of recycling procedures requires reliable and comprehensive information of the feedstock, which means reverse engineering in most cases since no information about for example cell chemistry is available.[^23^, ^24^] For example, elemental analysis of the starting material is needed to calculate recycling rates of targeted elements and possible impurities over the process. Furthermore, with possible LIB life times of up to >15 years, the return flow of spent LIBs will not represent state‐of‐the‐art (SOTA) materials, but various cell chemistries from more than a decade. Therefore, a reasonable value estimation of the present scrap requires elemental analysis of containing metal stoichiometries.^[1]^ For elemental analysis, inductively coupled plasma (ICP)‐based methods enable best sensitivity. Moreover, analytical methods like atomic absorption spectroscopy or X‐ray‐based methods like total reflection X‐ray fluorescence or energy dispersive X‐ray spectroscopy (EDX) can also give sufficient information and were successfully applied for LIB material characterization.[^25^, ^26^, ^27^, ^28^]

Beyond elemental analysis, speciation enables deeper insights, especially into present organic compounds. Analyses of the organic electrolyte, electrolyte additives, binder and their degradation species enable conclusions regarding cell aging conditions and material aging history. Moreover, possibly the recycling process interfering species, like binder polymer residues or potential dangers by hazardous species can be identified.^[23]^ Afterward, repeated analysis within the recycling procedure enables reliable process control by investigating the removal of these interferences. Especially, chromatography‐based investigations are well‐established for speciation of organic compounds in LIBs. After separation, mass spectrometric (MS) detection combines sensitivity and structural information for best compound identification.[^29^, ^30^]

Analytical investigations on LIBs were mainly applied to lab‐built and ‐aged cells or lab‐aged commercial cells. These samples secured information on materials, handling and aging to identify causal coherences between treatment and observed decomposition reactions. However, for more complex recycling material samples, studies on the transferability and adaptability of known methods are needed, as well as developments of new approaches for sample‐specific characterization.^[31]^ In this work, we report the application of analytical methods, previously established for laboratory aging and post mortem cell studies, to investigate unknown shredded LIB material from an industrial recycling process. Solely the positive electrode active material of the shredded cells was declared as LiNi_0.6_Co_0.2_Mn_0.2_O_2_ (NCM622) by the material supplier. Elemental analysis and speciation by chromatography‐based techniques were applied for detailed material characterization. Further, extraction‐ and pyrolysis‐based methods were conducted to maximize the accessible range of species. Based on the obtained information, the material history was accessed and starting points for analytical process control during subsequent recycling were highlighted.

## Experimental Section

**Chemicals**: The shredded LIB material was obtained from Duesenfeld (Germany), Acetonitrile (ACN) (>99.9 %) was purchased from VWR (USA) and dichloromethane (DCM) (99.8 %) from Merck (Germany). 1‐propanesulfonyl chloride (97 %) was obtained from Sigma Aldrich (Germany) and ethanol (EtOH) (96 %) was obtained from VWR. 1,3‐propanesultone (PS) (99 %) was purchased from Alfa Aesar (Germany).

**Extractions of volatile and soluble species for chromatographic analysis**: Solely dry shredded material was obtained. Therefore, extraction methods were applied to access electrolyte residues as well as decompositions species.

For analysis of volatile species, solid phase microextraction (SPME) was done with acrylate fibers in headspace mode with short (10 s) and long (600 s) sampling durations to preconcentrate main constituents and further detectable compounds, respectively. The solid sample was held at room temperature to prevent further aging by thermal decomposition during the sampling procedure. A SPME setup from CTC Analytics (Switzerland) controlled by the cycle composer software of the AOC 5000 autosampler (Shimadzu, Japan) was used. Further parameters were applied according to Horsthemke et al.^[32]^

For analysis of nonvolatile species, liquid extraction was performed. ACN was chosen as solvent, since it is typically used during sample preparation for liquid chromatography (LC) analysis and solves most literature known decomposition species. The pure shredded material (6.5 g) was transferred into a 50 mL Vial and 5 mL ACN were added. The mixture was intensively shaken for 5 min and filtered with a syringe filter (22 μm) to obtain a clear liquid solution that was analyzed by LC‐MS (undiluted) and ion chromatography‐conductivity detection (IC‐CD) (1/100 *v*/*v*). (Figure S1).

Further, the shredded material was extracted analogously with nonpolar DCM to solve organic carbonate residues and prevent conducting salt solvation for subsequent gas chromatography (GC)‐MS analysis with liquid injection.

**Sieving**: The shredded LIB material was separated into particle size fractions by analytical sieving.[^33^, ^34^] Sieving analysis was performed by Model HAVER EML 450 digital plus (HAVER & BOECKER, Germany) for 10 minutes using screens of 0.5, 1.0, 2.0, 3.15, 5.0, 8.0, 10.0, 12.5 and 16.0 mm. Therein, the black mass fraction <0.5 mm is an important material fraction concentrating mainly the liberated coating materials from the electrodes.^[35]^ Hence, the black mass fraction was further sieved at 0.063, 0.090, 0.100, and 0.315 mm.

## Analytical investigations

**Pyr**‐**GC**‐**MS**: For investigations with pyrolysis (Pyr)‐GC‐MS, a PY‐3030D pyrolyzer (Frontier Laboratories, Japan) was used. Measurements were conducted according to Stenzel et al.^[36]^ Adjusted pyrolysis temperatures of 200, 300 and 515 °C represented a compromise for simultaneous measurement of positive and negative electrode materials in the shredded material mixture, based on evolved gas analysis results in a previous study.^[36]^

**GC**‐**MS**: GC‐MS experiments were executed on a Shimadzu GCMS‐QP2010 Ultra with assembled AOC‐5000 Plus autosampler and a nonpolar Supelco SLB‐5 ms (30 m×0.25 mm. 0.25 μm; Sigma Aldrich) column. Further parameters were applied according to Horsthemke et al.^[32]^

GC investigations with high resolution (HR)MS detection were performed on a Q Exactive GC Orbitrap GC‐MS/MS system with a TRACE 1310 GC and a TriPlus RSH autosampler (all Thermo Fisher Scientific, USA). Experimental parameters were applied according to Peschel et al.^[37]^ and target analysis was performed based on extracted ion chromatograms (EICs) of measured accurate masses with a mass window of 5 ppm.

**LC**‐**MS**: For LC investigations with ion trap‐time of flight (IT‐TOF)‐MS detection, a Nexera X2 UHPLC system (Shimadzu) hyphenated to a LCMS‐IT‐TOF (Shimadzu) was used. Reversed‐phase (RP) chromatography was conducted on a ZORBAX SB−C18 column (100×2.1 mm, 1.8 μm; Agilent, USA) at 40 °C and a flow rate of 0.5 mL min^−1^. The analyte target list and further experimental parameters were applied according to Henschel et al.^[38]^

**IC**‐**CD**‐**MS**: IC investigations were performed on an 850 Professional IC (Metrohm, Switzerland) with conductivity detection (CD). For MS detection, the system was further hyphenated to the IT‐TOF‐MS. A Metrosep A Supp 7 column (250×4.0 mm, 5 μm; Metrohm) was used for isocratic anion separation at 65 °C and a flow rate of 0.7 mL min^−1^ was applied. The applied method is based on Kraft et al.^[39]^ and further parameters were applied according to Henschel et al.^[40]^

**ICP‐OES**: ICP‐optical emission spectroscopy (OES) measurements were performed using an ARCOS (Spectro Analytical Instruments, Germany) with an axial positioned plasma torch. For analysis, multiple emission lines were observed. All other parameters and sample preparations were applied according to Vortmann et al. and Evertz et al.[^41^, ^42^]

**SEM and EDX**: For scanning electron microscopy (SEM) and EDX analysis, material from a sieved fraction (0.5–1 mm) was optically presorted. Coppery and silvery colored flakes were separately attached to the sample trays. SEM measurements were performed by an Auriga electron microscope (Carl Zeiss Microscopy, Germany) with an accelerating voltage of 3 kV and EDX measurements were carried out with an accelerating voltage of 20 kV with an energy dispersive X‐ray detector (Oxford Instruments, United Kingdom).

## Results and Discussions

End‐of‐life LIBs obtained from an industrial shredding process were investigated to get insights into material composition. For first impressions of the inhomogeneous shredded material, optical presorting was performed. The material showed larger coppery and silvery colored flakes with attached black mass, different plastic pieces, remaining hard housing and black mass. (Figure S2) The optically presorted material was chosen for some experiments, as well as two sieved material fractions (0.5–1.0 mm and 0.100–0.315 mm).

**Figure 1:**
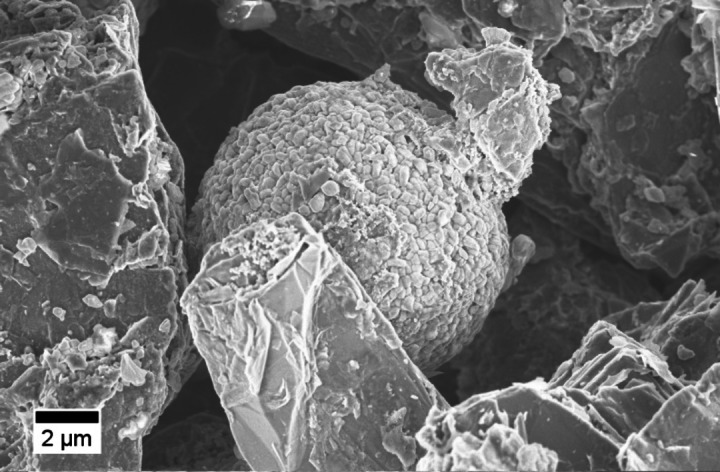
SEM image of a coppery colored flake (assumed as negative electrode originating) showing NCM particles in the graphite material after the shredding procedure.

More detailed optical impressions were obtained via SEM imaging. The SEM image of an optically presorted coppery colored flake (0.5–1.0 mm fraction, assumed as negative electrode origin) is shown in Figure 1. The SEM image illustrates partially mixed positive (round shaped NCM) and negative electrode (graphite flakes) material also on particle level. In contrast to dissembled cells, major material inhomogeneities have to be considered for analytical sample complexity, but also for material recycling.

## Analysis of the elemental composition

To analyze the elemental composition, representative samples of the shredded material were measured via ICP‐OES after microwave assisted digestion (threefold determination). Main focus was to reconstruct the active material stoichiometry of the positive electrode. Conclusions regarding current collector metals, electrolyte constituents and binder materials were also drawn.

The Ni/Co/Mn ratio was determined as 0.58/0.21/0.21. The cumulated proportion of the positive electrode transition metals was 24.03 (±0.69) wt %. Further quantified elements were Li (2.77 (±0.08) wt %), Al (5.14 (±1.35) wt %), Cu (7.61 (±1.68) wt %), P (0.77 (±0.03) wt %), Na (0.15 (±0.01) wt %), S (0.14 (±0.01) wt %), Mg (0.38 (±0.01) wt %) and Zr (0.17 (±0.01) wt %). The measured stoichiometry of Ni_0.6_Co_0.2_Mn_0.2_ agreed with the limited information obtained by the material supplier. It has to been stated, that mixtures of NCM or additional LiNi_x_Co_y_AlO_2_ (NCA) stoichiometries could make this analysis more complicated. If the ICP‐OES/MS results hint at a mixture of NCM and/or NCA materials, for example single particle investigations, recently introduced by Kröger et al.,^[43]^ could give further insights regarding mixed stoichiometries and varying active materials.

The quantified Li proportion can originate from the NCM material, as well as from the conducting salt. The common commercially used conducting salt is LiPF_6_, whose occurrence was analyzed in more detail by IC‐CD analysis. Further, copper and aluminum were identified, commonly applied as current collector materials for the negative and positive electrode, respectively. This conclusion was further proven by a combination of SEM and EDX measurements of optically presorted flakes. The identified proportion of sodium indicated the use of carboxymethyl cellulose (CMC) as a binder material, which is commonly applied as sodium salt.[^44^, ^45^] Binder polymers were further analyzed by Pyr‐GC‐MS. Sulfur could originate from additionally applied sulfur containing conducting salt anions like bis((trifluoromethyl)sulfonyl)imide (TFSI^−^) or bis(fluorosulfonyl)imide (FSI^−^) which was further investigated by IC‐CD‐IT‐TOF‐MS, and from application of sulfur containing electrolyte additives, as further investigated by Pyr‐GC‐MS and GC‐HRMS.

In addition to representative samples of the shredded material, also sieved fractions (0.5–1 mm and 0.100–0.315 mm) were analyzed by ICP‐OES to determine changes of elemental composition caused by the choice of sample constitution. Significant differences were observed for the current collector metals. The 0.5–1 mm fraction contained higher Al (10.19 (±0.60) wt %) and Cu (19.91 (±0.95) wt %) contents with lower deviations for multiple digestions. The fraction (see Figure S2) mainly consisted of small, coated electrode flakes reflected by the higher current collector metal contents in the ICP‐OES measurements. In contrast, the fine (0.100–0.315 mm) fraction showed lower contents compared to the representative unsieved sample with 2.39. (±0.05) wt % and 1.98 (±0.95) wt % for Al and Cu, respectively. The significant differences illustrate inhomogeneous sample constitution after shredding and relevance of representative sample choice, to obtain reliable insights into elemental material composition.

For recycling purposes, elemental composition analysis of starting material with unknown history is inevitable. The value of unknown LIB scrap highly depends on the applied positive electrode material due to different material values of Ni, Co and Mn.^[8]^ Accordingly, higher cobalt contents as applied in LiNi_0.33_C_0.33_M_0.33_O_2_ or LiCoO_2_ materials could account for higher scrap prices compared to NCM622‐based material. Not only with the return flow of LIB chemistries from multiple decades, but also with continuous reduction of inactive material contents for improved energy densities, elemental value of the scrap varies.^[1]^ Moreover, identification of mixed positive electrode materials is relevant for robust hydrometallurgical process control, for example by consideration of LiFePO_4_ contents.^[46]^

## Analysis of organic compounds

Elemental analysis was informative for recycling valuable estimation by identification and quantification of positive electrode‐based (transition) metals and current collector materials. However, only 41.16 (±3.88) wt % of the overall sample mass was dedicated to the quantified elements. Further, graphite, applied as negative electrode active material, was identified by SEM‐EDX imaging. Anyhow, fluorine species and further organic residues are present in the shredded material and knowledge on these is valuable for tailored treatment. Further sample characterization can be obtained via speciation of the organic substances. Therefore, chromatographic techniques mainly coupled to MS detection were applied.^[29]^ Moreover, pyrolysis, preconcentration and extraction methods were occupied.

## Pyr‐GC‐MS investigations

**Figure 2:**
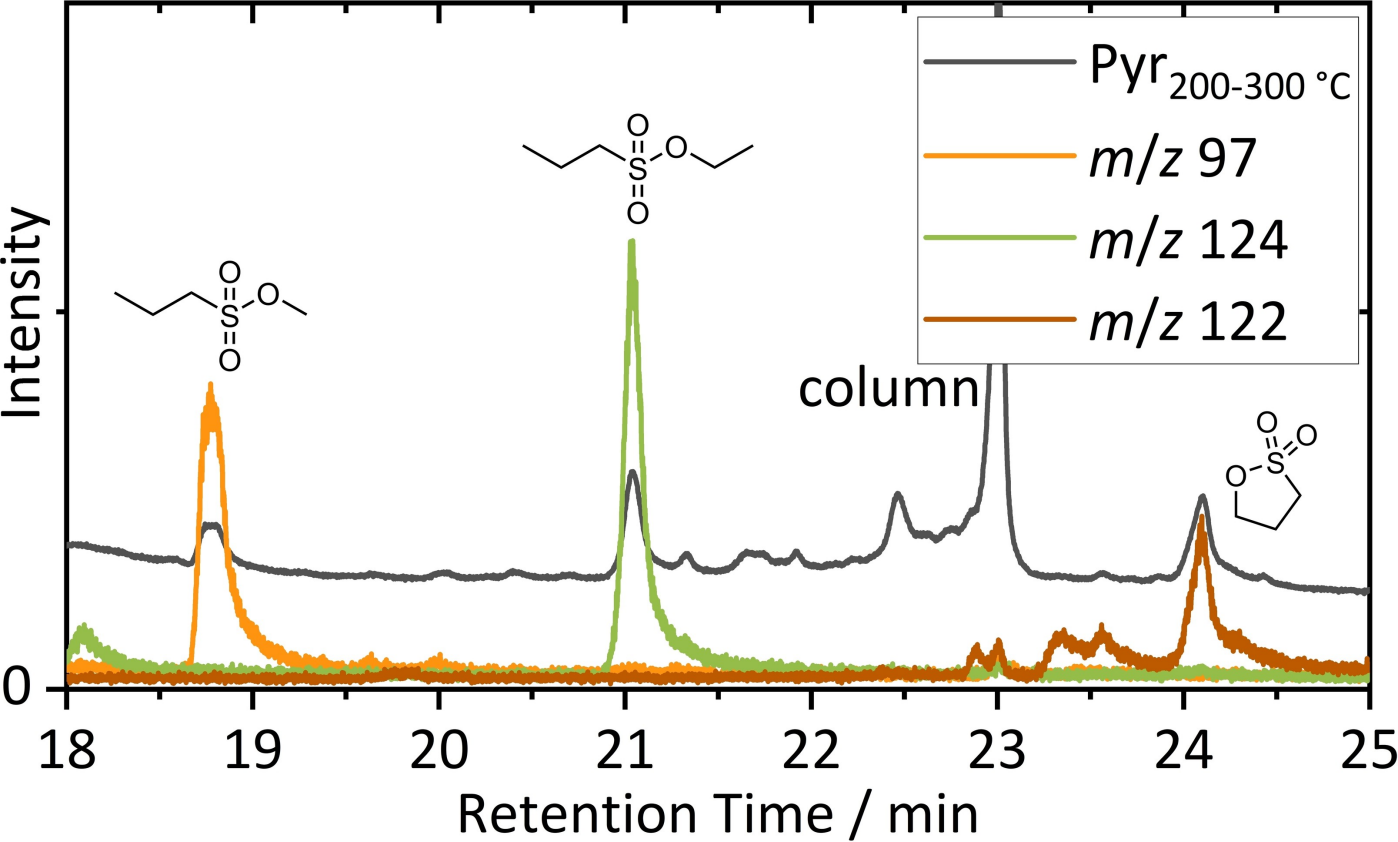
Identification of MPS, EPS and PS by Pyr‐GC‐MS. For clarity, EIC intensities are magnified by factor 100. Intensive column caused peaks can be explained by unavoidable and harmful PF_6_^−^ pyrolysis.

Pyr‐GC‐MS was applied to analyze polymeric binder residues in the shredded material. After shredding, electrode materials are randomly mixed and separate analysis of positive and negative electrode materials as described in literature was not practicable.^[36]^ For easy sample handling, first a random sample of a sieved fraction (0.1–0.315 mm) was analyzed. The pyrograms obtained after pyrolysis at 200 and 300 °C, mainly showing literature known electrolyte residues and electrolyte decomposition products, are shown in the Supporting Information (Figure S3). Identification of electrolyte residues and decomposition species will be discussed based on (SPME)‐GC‐MS results, later. Significant amounts of 1‐propanesulfonic acid ethyl ester (EPS) and PS were identified after pyrolysis at a temperature of 300 °C by data base comparisons (NIST 11 scores >94 %). (Figure S4) To prove these findings, additionally optically presorted coppery colored flakes were analyzed. An excerpt of the overlay of the TIC and by factor 100 magnified EICs of marker fragment ions is depicted in Figure 2. Also, 1‐propanesulfonic acid methyl ester (MPS) was identified (NIST 11 score 92 %) whose identification suffered from peak overlapping caused by the sample complexity in previous measurement. The obtained background subtracted GC‐single quadrupole (SQ)‐MS mass spectra of the three identified sulfur containing analytes are shown in the Supporting Information (Figure S6 and S7). Target analysis of EPS and PS was also performed by GC‐HRMS and will be discussed in a following section.

MPS, EPS and PS were identified at a pyrolysis temperature of 300 °C. Regarding PS, the M^+^ ion with *m*/*z* 122 was identified, due to limited fragmentation behavior on the SQ‐MS system. PS is applied as a film forming additive and ring opening reactivity during cell formation was described. After ring opening of PS, lithium alkyl/alkenyl sulfonates are formed that were reported to improve the lithium ion conductivity of the SEI for graphite‐based negative electrodes.[^47^, ^48^, ^49^, ^50^]

Relating to reversed engineering approaches, PS can be used alone or in combination with further substances such as fluoroethylene carbonate (FEC) and vinylene carbonate (VC) as film former.^[47]^ No clear evidence for either of them was detected, as discussed in a following section. Concerning recycling purposes of the shredded LIB material, PS exposition at elevated temperatures has to be considered. PS suffers from major toxicity and also volatile derivates should be treated as possible dangers.[^51^, ^52^] Further, degradation reactions of PS resulting in toxic and highly volatile SO_2_ or H_2_S are conceivable at higher temperatures, but were not observed in these experiments. Besides hazard potential, sulfur containing additives represent a further hetero atom containing specie, relevant for example for hydrometallurgical treatment.

**Figure 3:**
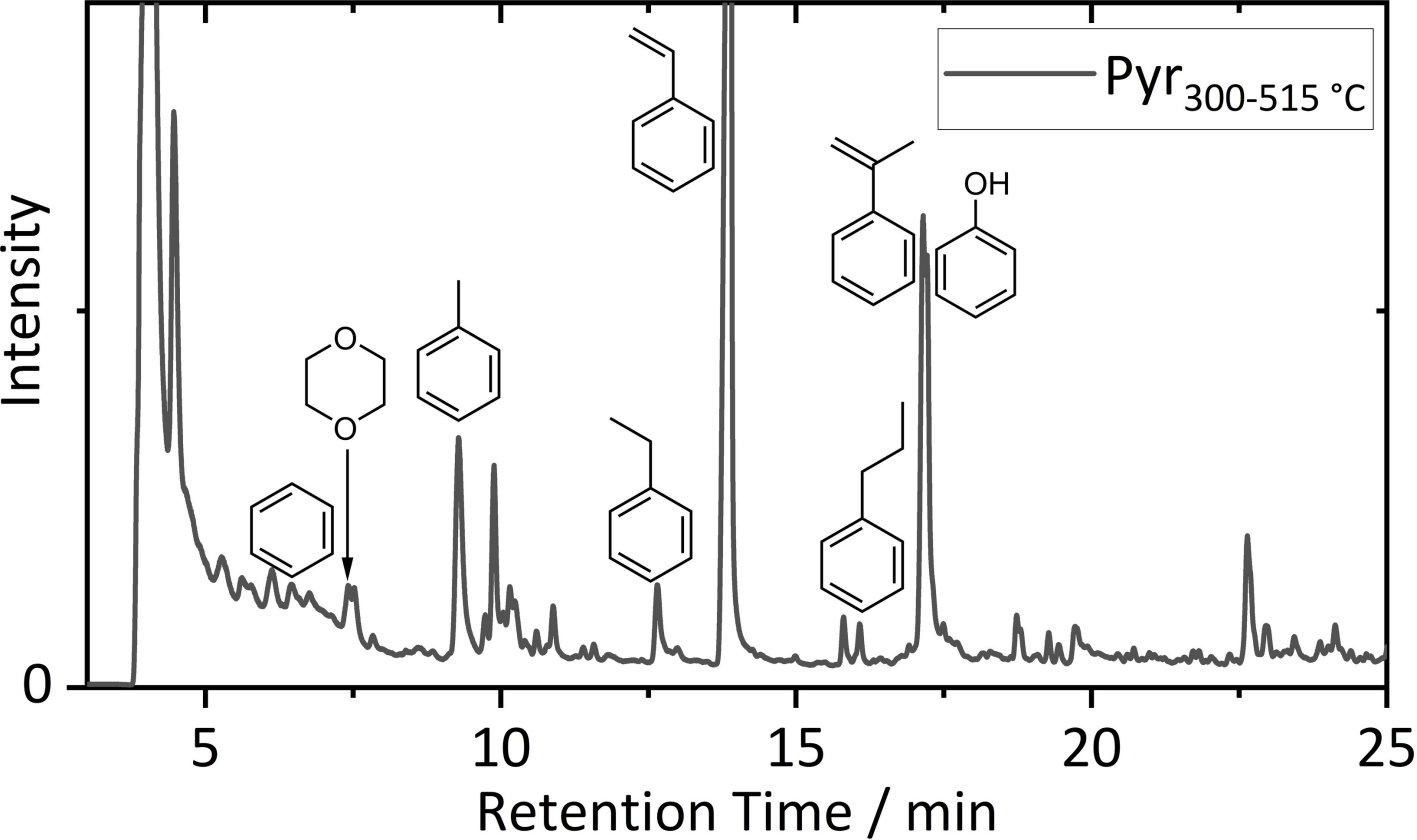
Pyrogram of a sieved fraction (0.1–0.315 mm) of the shredded LIB material at a pyrolysis temperature of 515 °C. Typical binder originating structures are depicted according to literature based on NIST 11 database identification.^[36]^

The obtained pyrogram at a pyrolysis temperature of 515 °C is depicted in Figure 3. The sample complexity resulted in complex pyrograms with peak overlapping.

Target analysis of previously reported markers of typical binder materials was conducted.[^36^, ^53^] Especially the benzylic fingerprint of styrene‐butadiene rubber (SBR) with benzene (6.45 min), toluene (9.28 min), ethylbenzene (12.65 min), styrene (13.80 min), propylbenzene (16.08 min), prop‐1‐en‐2‐ylbenzene (17.14 min) and phenol (17.22 min) was identified, concluding SBR as an applied binder material. SBR is usually applied in combination with CMC in aqueous processed SOTA graphite‐based negative electrodes.[^54^, ^55^] Detection of Na by ICP‐OES already hinted at CMC usage, but only 1,4‐dioxane (7.40 min) was found by Pyr‐GC‐MS, probably originating from CMC.^[36]^ However, concluding a mixture of CMC with SBR as main negative electrode binder material was reasonable. For identification of the positive electrode binder material, hints of the SOTA material polyvinylidene difluoride (PVdF) were found at a pyrolysis temperature of 515 °C. The EIC of *m*/*z* 64, reported as C_2_F_2_H_2_ in literature, is shown in the Supporting Information (Figure S5), but suffered from major peak overlapping with further highly volatile species at short retention times in the total ion chromatogram (TIC).[^36^, ^53^]

For recycling of the active materials, organic compounds like carbonate or binder residues are usually removed.[^56^, ^57^] Investigations by Pyr‐GC‐MS were proven as a powerful tool to identify electrolyte and binder residues in the inhomogeneous shredded material. The parallel identification of electrolyte (additive) residues, decomposition species and binder materials enabled fast and broad‐ranging screening on organic compounds. Further, based on Pyr‐GC‐MS investigations, also polymer electrolytes or separators could be investigated.

Subsequent material treatment can be tailored based on these investigations, for example thermal treatment temperatures can be adjusted based on identified materials and furthermore, this paves the way for customized quality control of process steps aiming at organic compound removal during material recycling. Target analysis by Pyr‐GC‐MS after thermal, mechanical and/or chemical treatment could be performed to control successful removal of the species.

## GC‐MS investigations

**Figure 4:**
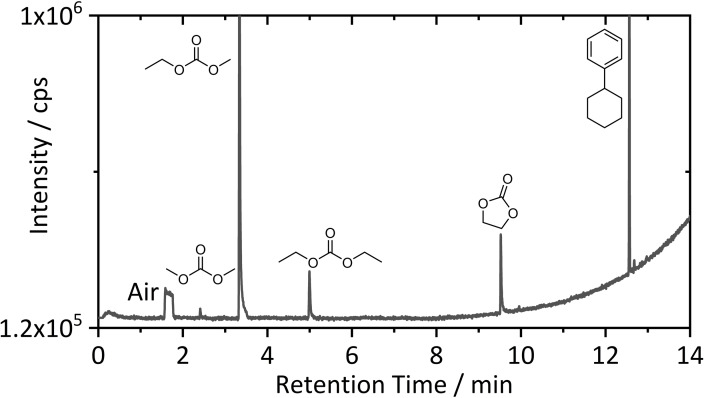
SPME‐GC‐MS chromatogram of a solid sample of shredded LIBs after preconcentration for 10 s. Structures of identified main constituents are depicted.

For more detailed insights into species with significant vapor pressure at room temperature, SPME‐GC‐MS was performed. SPME‐GC‐MS enables fast screening also of dry materials without any sample treatment.^[32]^ The headspace above a random solid sample was analyzed without heating to avoid ongoing decomposition. The obtained SPME‐GC‐MS chromatogram after preconcentration for 10 s is depicted in Figure 4.

Five main peaks representing the analyzed sample and an additional peak caused by detected air (1.56–1.77 min) were found. The observed linear carbonates dimethyl carbonate (DMC) (2.41 min), ethyl methyl carbonate (EMC) (3.34 min) and diethyl carbonate (DEC) (4.99 min) are typical SOTA LIB electrolyte solvent molecules. The electrolyte(s) applied in the investigated cells might consisted of mixtures of these linear carbonates, but it seems more plausible, that the symmetric linear carbonates were formed by transesterification reactions of EMC and the pristine electrolyte formulation was solely EMC‐based.^[58]^ For this case, the relatively low degree of transesterification also indicated the application of an interphase film forming additive. PS was identified, but further commercially applied film forming additives such as FEC and VC were not observed.^[59]^ However, their complete consumption cannot be excluded.

The SOTA electrolyte solvent ethylene carbonate (EC) (9.52 min) was also detected. Besides linear and cyclic carbonates, cyclohexylbenzene (CHB, 12.56 min) was detectable after a short extraction time. The large peak compared to the carbonate‐based solvent molecules is caused by the good ionization efficiency of the aromatic ring structure. CHB is known as an overcharge protective shuttle additive and was found in aged electrolytes of LIBs for high power applications in (P)HEV with quantities in the low percent range.[^40^, ^60^] In conclusion, the identification of CHB indicated a possible lifetime application of the analyzed LIB material.

For analysis of lower concentrated volatile species, the sample was also extracted for 600 s. Magnified sections of the obtained chromatogram are depicted in the Supporting Information (Figure S8). Besides the previously described substances, further literature known decomposition species like C_3/4_ carbonates,[^61^, ^62^] dimethyl (DMDOHC)‐ ethylmethyl (EMDOHC)‐ and diethyl‐2,5‐dioxahexane dicarboxylate (DEDOHC),[^58^, ^63^] and applied electrolyte components like biphenyl (BP)^[64]^ and propylene carbonate (PC)^[65]^ were detected. Carbonates with elongated alkyl chains and oligo carbonates are typical electrolyte decomposition species after electrochemical (=cyclic) aging.[^62^, ^63^] Possible explanations for the occurrence of PC and BP range from combined application with other applied compounds (e. g. BP and CHB), over impurities (e. g. BP in CHB), to simultaneous shredding of different cells or carry over contaminations during the shredding process.[^40^, ^64^]

SPME enabled fast and simple characterization of highly volatile substances. Anyhow, for sampling at room temperature, analytes with low vapor pressures suffer from sensitivity discrimination due to lower headspace extraction yields. Therefore, liquid extraction with a nonpolar solvent (DCM) was performed to enable liquid injection into the GC system despite dry sample material. DCM was chosen as it is proven to minimize conducting salt injection based on low LiPF_6_ solubility.^[66]^ The resulting GC‐MS chromatogram is shown in the Supporting Information (Figure S9). In addition to previously performed SPME‐GC‐MS analysis, further benzylic species were identified by NIST 11 database comparisons of background subtracted mass spectra (scores >90 %). Moreover, adiponitrile (ADN) was detected, also reported as a high voltage‐compatible and high flashpoint component in LIB electrolytes.[^67^, ^68^] Solely qualitative data hindered conclusions regarding reversed engineering, but identification illustrated possible material complexity after industrial shredding with high probabilities of cross contaminations.

**Figure 5:**
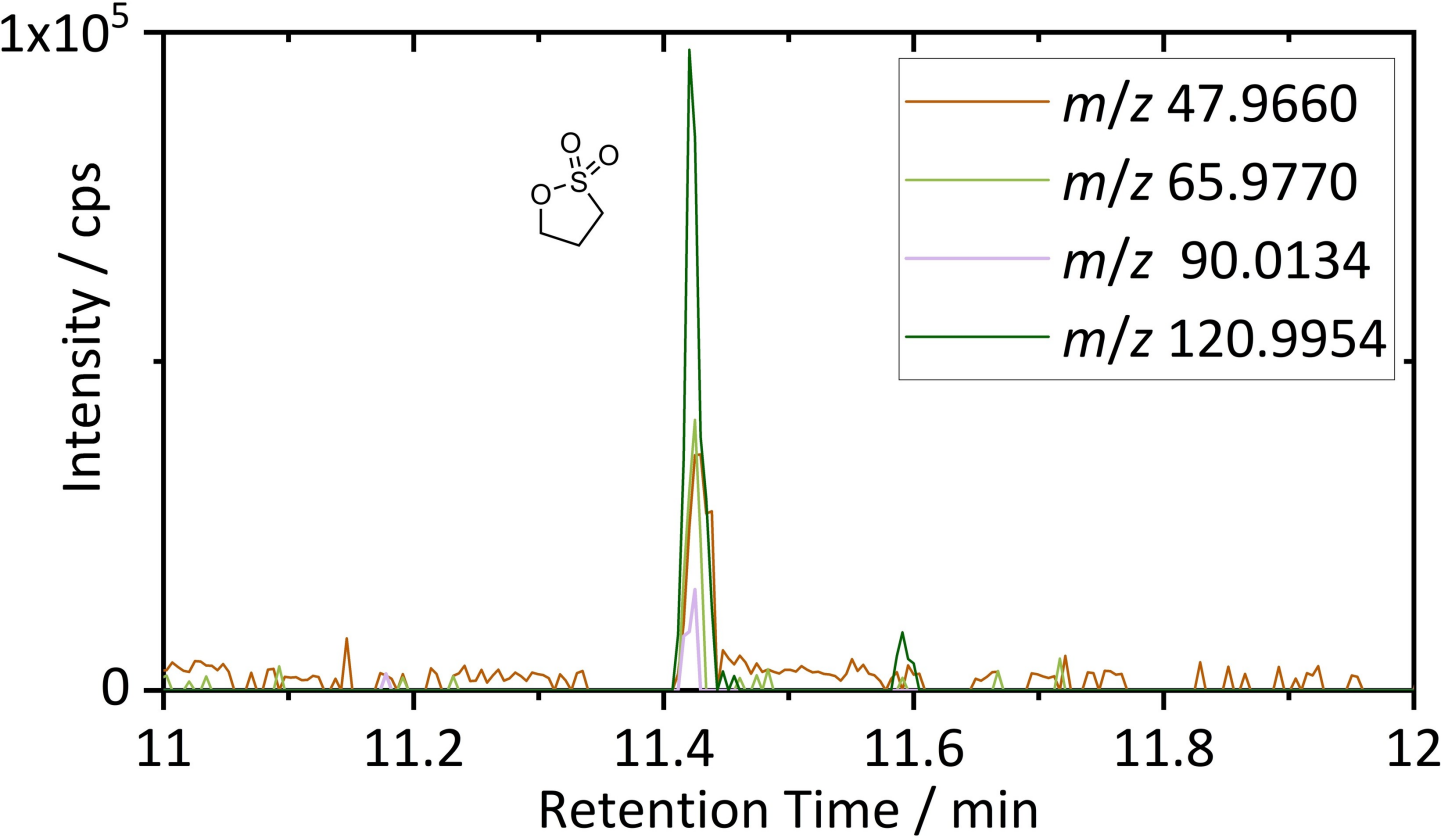
GC‐HRMS chromatogram of PS by target analysis. Mass traces were chosen based on the measured spectrum of a reference material.

For improved sensitivity and selectivity, the DCM extract was also investigated by GC‐HRMS. Structures, previously identified based on GC‐SQ‐MS database comparisons, were confirmed utilizing accurate mass capabilities. Moreover, pyrolysis findings on PS were investigated. A commercially available PS standard was investigated to obtain exact knowledge on retention and fragmentation behavior on the GC‐HRMS system. Based on this data, target analysis by EICs of characteristic sulfur containing fragment ions was conducted. The overlay of the characteristic EICs is depicted in Figure 5. For chromatographic data of the PS standard material and the obtained GC‐HRMS mass spectrum, the reader is kindly referred to the Supporting Information (Figure S10).

Target analysis of PS (11.39 min) resulted in very low intensities of the chosen sulfur containing marker fragment ions at the contemplable retention time. The identification of PS was within the limits of detection. Combining Pyr‐GC‐MS and GC‐HRMS results, PS and propyl sulfonates have to be considered as occurring sulfur containing species in the shredded material. EPS was synthesized for reliable identification by retention time and characteristic fragment ions by means of GC‐HRMS. The resulting chromatogram of the reaction mixture, the GC‐HRMS mass spectrum and identification of EPS in the DCM extract is depicted in the Supporting Information (Figure S11). Identification of EPS in the DCM extract proved, that the esterification of the alkyl sulfonate also occurred without pyrolysis and therefore, is concluded as a PS aging marker. However, this will be investigated in separate works with aged, but unshredded PS containing cells with precise knowledge of electrolyte, active material and aging conditions.

The combined results from the performed GC‐MS measurements underline the value of comprehensive analytical investigations for both, highly informative and highly reliable material characterization, even for the same chromatographic technique.

## LC‐MS and IC‐CD(‐MS) investigations

**Figure 6:**
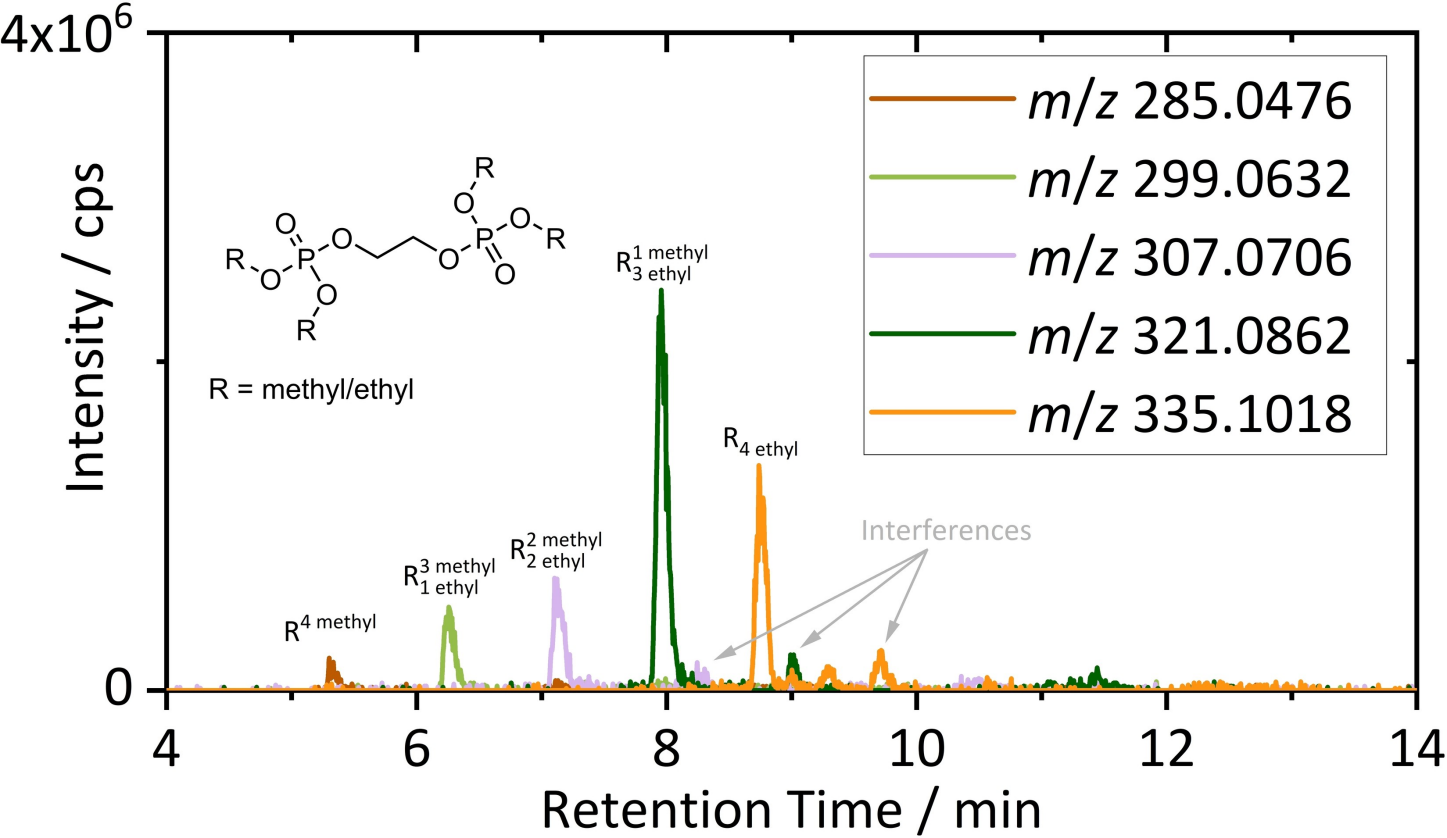
Overlay of EICs of oligo phosphate adducts as exemplarily chosen target molecules in a RPLC‐IT‐TOF‐MS chromatogram obtain from the ACN extract of the shredded material. Extracted accurate masses were chosen according to adducts observed by Henschel et al.^[38]^

For LC‐MS target analysis of electrolyte decomposition species, the shredded material was extracted with ACN. As for GC analysis, data evaluation was simplified by previously defined target marker molecules.^[38]^ Target analysis by EICs was performed based on literature known species and adduct formation. Among others, oligo carbonate, phosphate carbonate and oligo phosphate species were detected. Further, ether oligomers and carbonate ether co‐oligomers, described as thermal strain markers, were identified. Altogether, more than 50 species were identified within the observed mass range, solely based on the target lists introduced by Henschel et al.^[38]^ (Table S1–S4) Exemplarily, Figure 6 shows an overlay of EICs of characteristic adducts formed for diphosphates with varying alkylation. ((Me)_4_→(Et)_4_)

In previous studies oligo phosphates were found in EMC and VC containing electrolytes after >1000 cycles, but not in film forming additive free electrolytes or solely after cell formation. Based on this, these species were described as possible VC marker molecules.^[38]^ Afterward, the detection in DEC‐based electrolytes after >500 cycles was also described with low intensities.^[63]^ For DEC‐based electrolytes, it has to be considered, that only tetra ethyl species are formed, and therefore, the overall detection limits for the substance class suffer from statistical effects depending on the linear carbonate. Nevertheless, conclusions related to cell operating and aging history of the analyzed material were enabled, based on the detection of these species. Obviously, the material underwent long‐term cyclic aging before shredding. Moreover, detection of significant intensities of oligo phosphates correlated with Pyr‐GC‐MS and GC‐HRMS indications on the usage of at least one film forming additive. The identification of thermal strain markers could not be clearly assigned to conditions during cycling, as thermal stress during the shredding and electrolyte removal processes was also reasonable.

**Figure 7:**
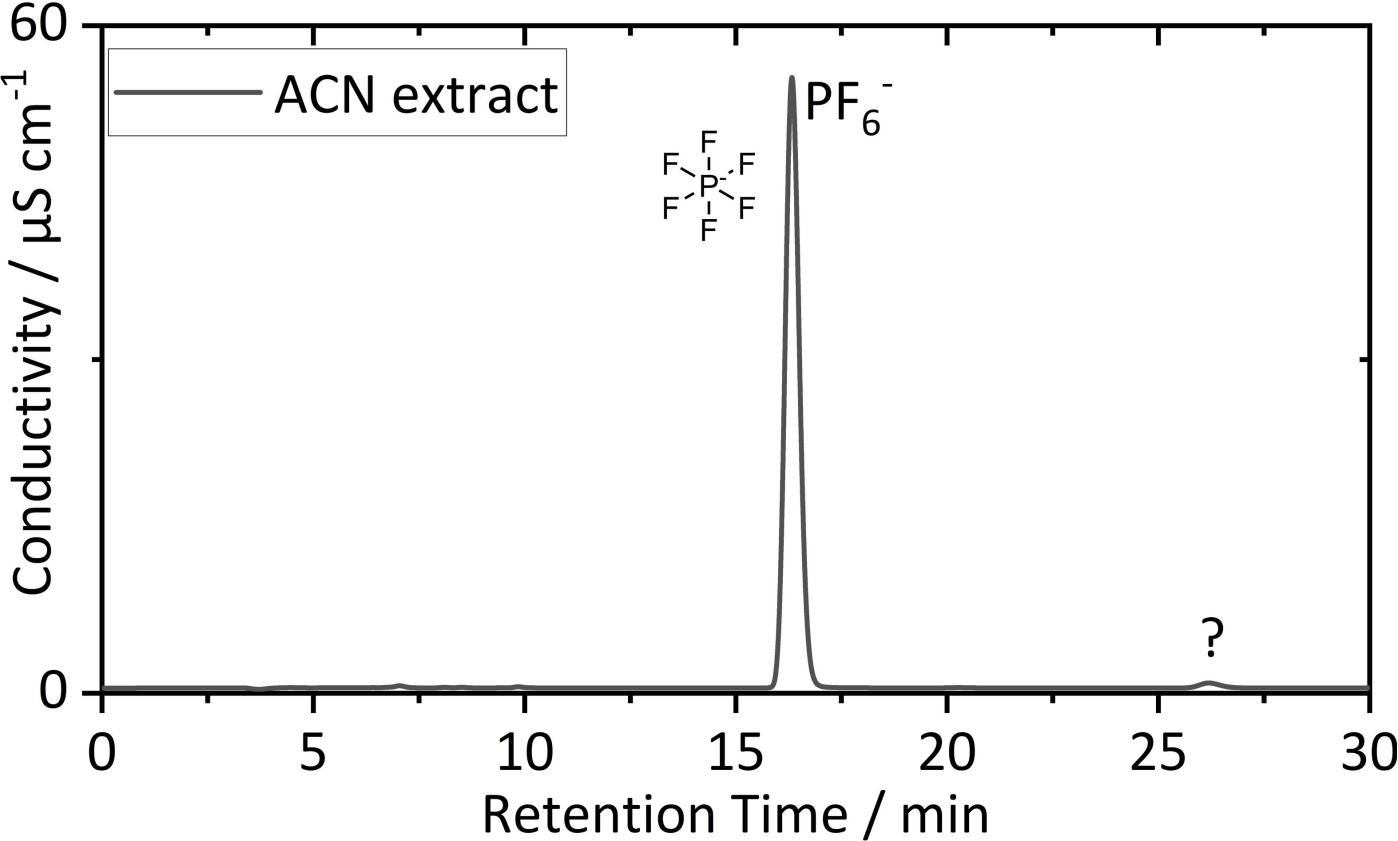
IC‐CD chromatogram obtained from the ACN extract after dilution (1/100, *v*/*v*). PF_6_^−^ was found as the dominating anionic specie.

In addition to RPLC separation, IC was conducted to get insights into detectable conducting salt anions and possible anionic decomposition species. The IC‐CD chromatogram obtained from the diluted (1/100 *v*/*v*) ACN extract is depicted in Figure 7.

**Figure 8:**
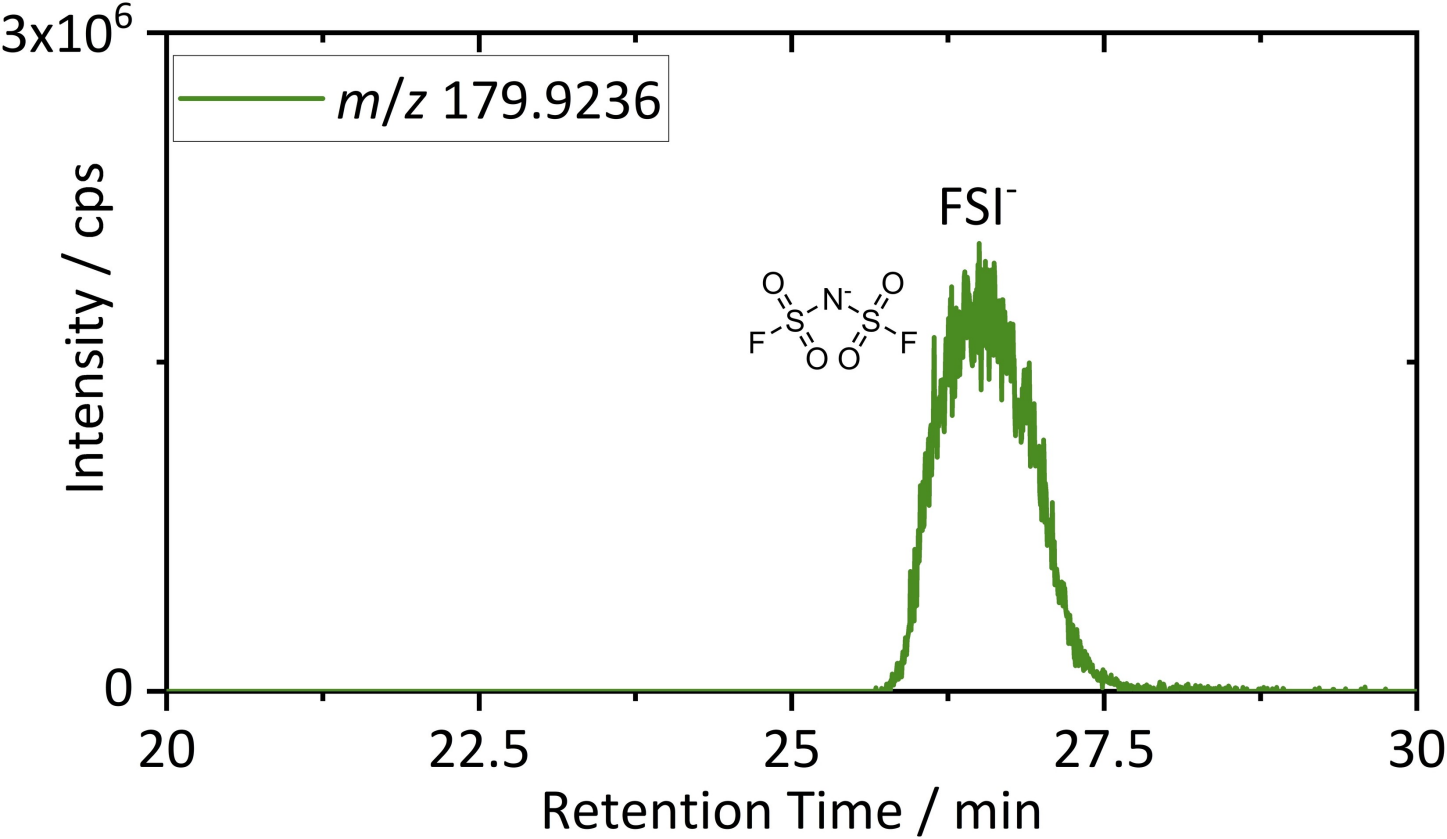
EIC of an IC‐IT‐TOF‐MS chromatogram for identification of FSI^−^.

Qualitative IC‐CD measurement proved PF_6_^−^ (16.3 min) as the dominating conducting salt anion, which is in line with the literature.[^69^, ^70^] A further peak with a retention time of 26.5 min was detected. Identification was performed via HRMS detection. The background subtracted mass spectrum of the peak at 26.5 min is shown in the Supporting Information (Figure S12). The EIC of the M^−^ ion is depicted in Figure 8.

The measured M^−^ ion with *m*/*z* 179.9236 belongs to FSI^−,^ which is also applied as conducting salt anion in LIBs.[^70^, ^71^] The origin of FSI^−^ could not be clearly determined. Application as a co‐conducting ion or cross contamination during shredding were possible explanations. Nevertheless, the identification represents another example of the complexity of the obtained recycling starting material. Anionic decomposition species like PF_6_^−^ hydrolysis products were not detected. Reasons could be their absence or low extraction yields via ACN. For recycling of the conducting salt anions, detection of significant amounts of undecomposed PF_6_^−^ after shredding and rough electrolyte removal paves the way for possible early‐stage recovery of the conducting salt via extraction methods. However, reproducibility and recovery rates of the performed solvent extraction require further evaluation.

## Conclusions

In this study, inhomogeneous shredded LIB material from an industrial recycling process was analyzed in detail. Comprehensive application of a wide range of analytical methods enabled deep understanding of elemental composition and present organic species. On the one hand, quantitative elemental analysis of material was informative for both, elemental recycling value and reversed engineering approaches. To illustrate sample inhomogeneities after shredding, sieved fractions of the shredded material were also analyzed, showing significantly different Al and Cu contents. On the other hand, organic speciation via chromatography‐based methods was applied and conclusions regarding electrolyte and binder aging history were drawn. Reasonable pristine materials were discussed and long‐term cyclic aging of the material was proven by different examples. Furthermore, obtained data enabled evaluation of challenges for recycling purposes like safety aspects and process interfering sulfur containing species. Thereby, comprehensive analysis combined the advantages of gas and liquid chromatography, as well as extraction and pyrolysis methods for maximized information output.

Performed comprehensive analysis proved demand and capabilities of analytical methods for characterization of shredded LIB material. Despite material complexity and lack of information on material history, deep understanding of the present recycling material can be obtained. Beyond that, approaches for ensuing recycling product as well as process control were pointed out based on the same analytical methods.

## Conflict of interest

The authors declare no conflict of interest.

1

## Supporting information

As a service to our authors and readers, this journal provides supporting information supplied by the authors. Such materials are peer reviewed and may be re‐organized for online delivery, but are not copy‐edited or typeset. Technical support issues arising from supporting information (other than missing files) should be addressed to the authors.

Supporting Information

## Data Availability

Research data are not shared.
