# Supplementary material for: Comprehensive Characterization of Shredded Lithium‐Ion Battery Recycling Material
Source: Chemistry. 2022 Mar 14;28(22):e202200485. doi: 10.1002/chem.202200485 (PMC9311206; doi:10.1002/chem.202200485)
Supplement: Supplementary file 1 — Supporting Information [file CHEM-28-0-s001.pdf]

Chemistry–A European Journal

Supporting Information

Comprehensive Characterization of Shredded Lithium-Ion Battery Recycling Material

Christoph Peschel, Stefan van Wickeren, Yves Preibisch, Verena Naber, Denis Werner, Lars Frankenstein, Fabian Horsthemke, Urs Peuker, Martin Winter, and Sascha Nowak*

Solvent Extraction

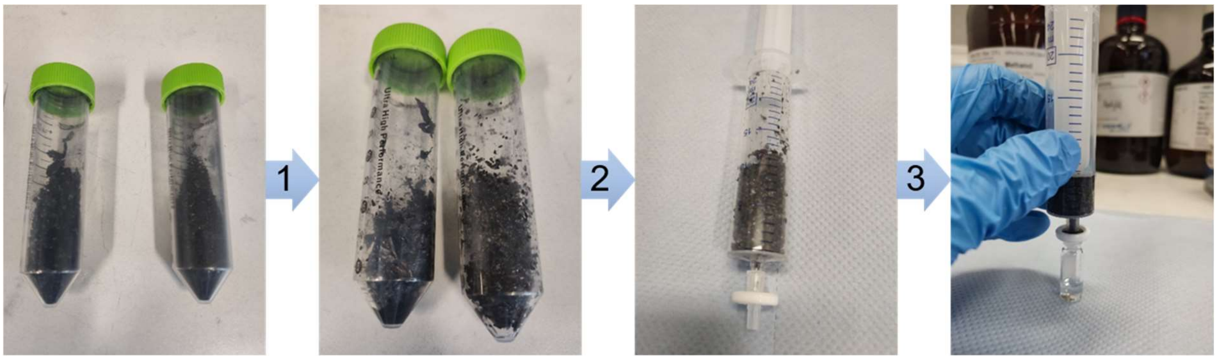

Figure S1: Visualization of the material extraction. The pure shredded material was transferred to a 50 mL vial. 5 mL of solvent were added (**1**). After intensive shaking, the mixture was transferred to a syringe (**2**) and filtered with a syringe filter (22 µm) to obtain a clear liquid solution (**3**).

Photo

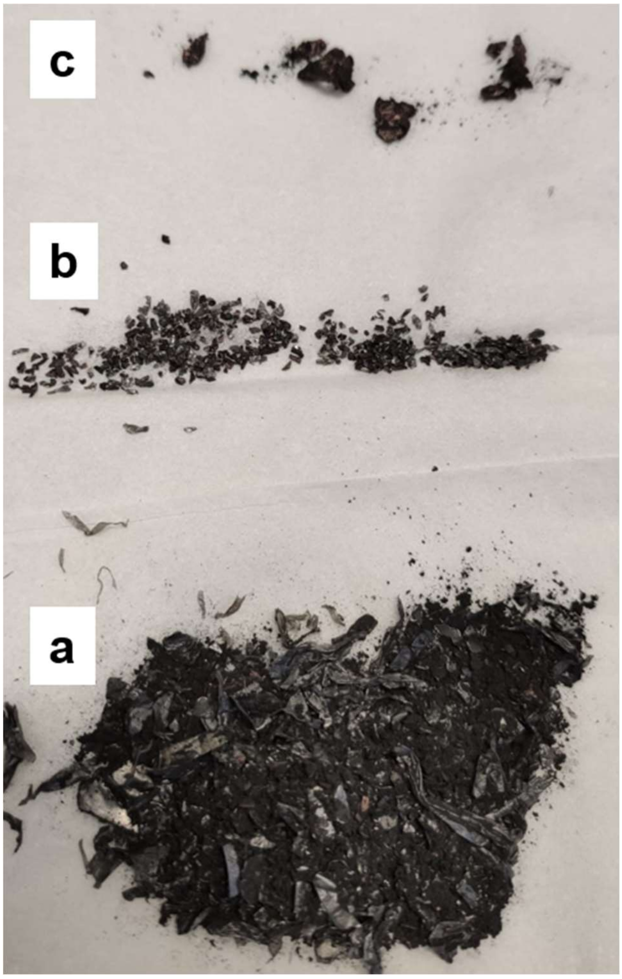

Figure S2: Photo of the analyzed shredded material as obtained (**a**), after sieving (0.5-1.0 mm, **b**) and presorted coppery colored flakes (**c**).

Pyr-GC-MS

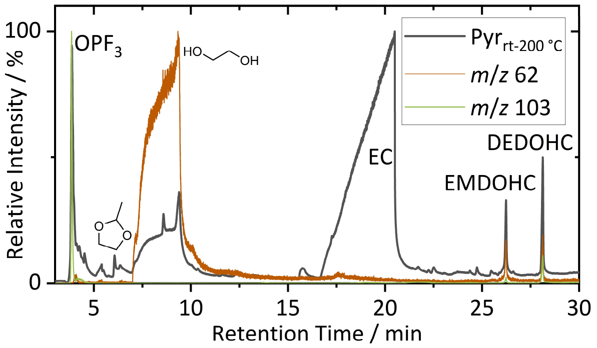

Figure S3: Pyrogram of a sieved fraction (0.100-0.315 mm) of the shredded LIB material at a pyrolysis temperature of 200 °C. EICs with *m*/*z* 62 and 103 are depicted for identification of ethylene glycol and OPF_3, respectively. Further, EC and typical electrolyte decomposition species were identified.

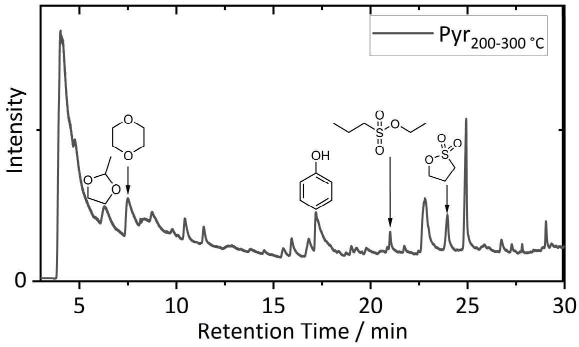

Figure S4: Pyrogram of a sieved fraction (0.100-0.315 mm) of the shredded LIB material at a pyrolysis temperature of 300 °C. Among others, 1,4-dioxane, phenol EPS and PS were identified.

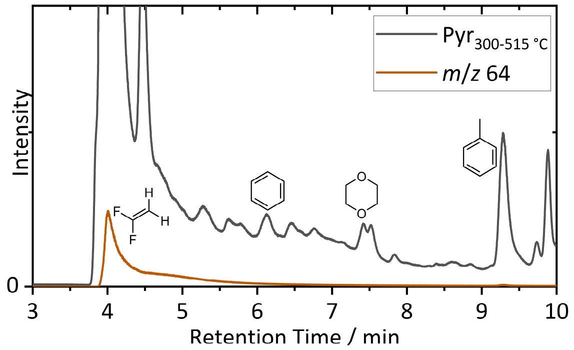

Figure S5: EIC of *m*/*z* 64 reported as $C_2F_2H_2$ in literature. The detection hinted at PVdF as applied positive electrode binder material.[1,2]

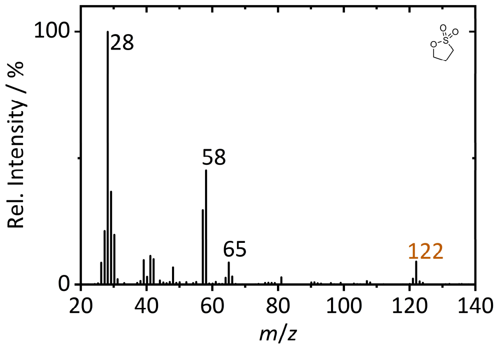

Figure S6: Measured background subtracted mass spectrum PS. The M^+ ion with *m*/*z* 122 was exemplarily chosen for identification in Figure 2.

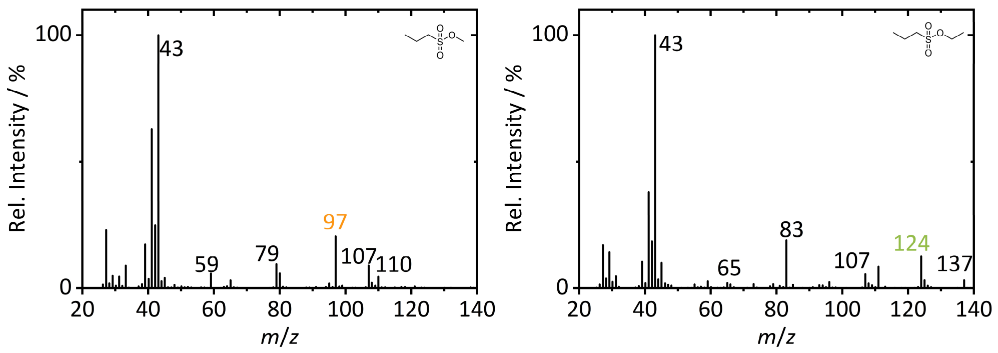

Figure S7: Measured background subtracted mass spectra of MPS (l.) and EPS (r.). The fragment ions with *m*/*z* 97 and 124 were chosen for identification in Figure 2.

SPME-GC-MS

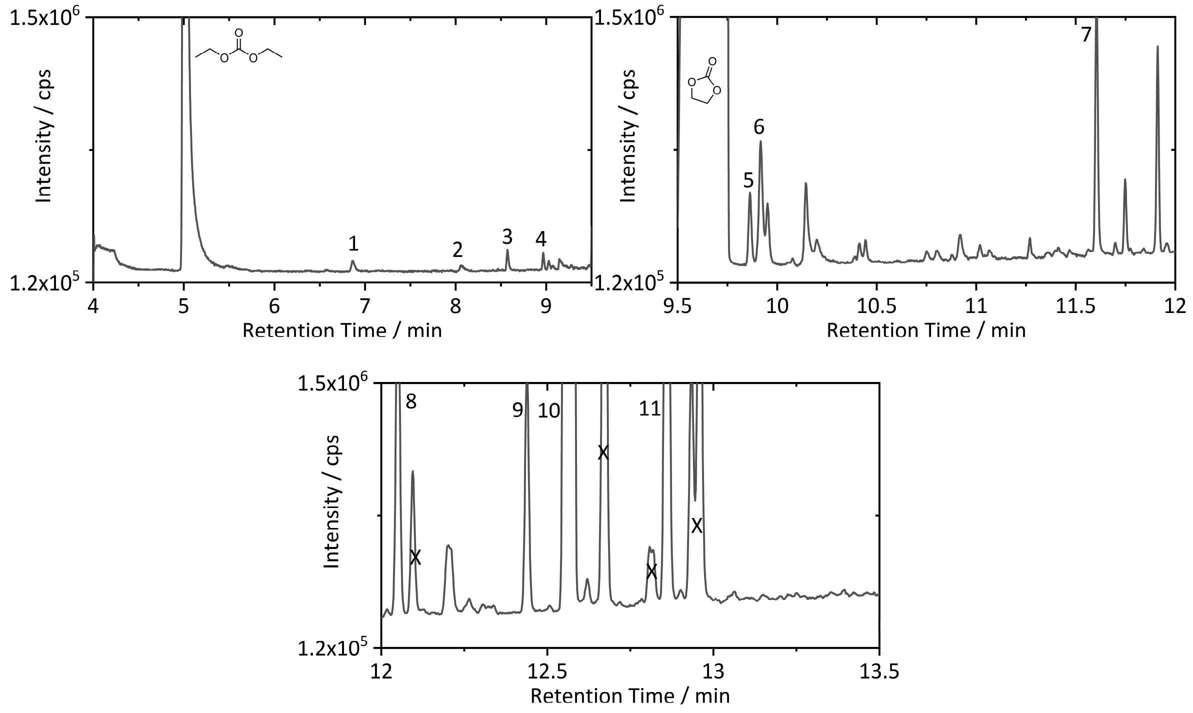

Figure S8: SPME-GC-MS chromatogram of a solid sample of shredded LIBs after preconcentration for 600 s. For illustrative reasons the chromatogram is divided in 3 sections. Species were identified based on NIST11 database comparisons as follows: **1**: *sec*-butyl methyl carbonate (*s*BMC, 6.86 min), **2**: ethyl propyl carbonate (EPC, 8.06 min); **3**: *n*-butyl methyl carbonate (BMC, 8.57 min). **4**: *sec*-butyl ethyl carbonate (BEC, 8.96 min), **5**: *n*-butyl ethyl carbonate (BEC, 9.86 min), **6**: PC (9.91 min), **7**: DMDOHC (11.60 min), **8**: EMDOHC (12.05 min), **9**: DEDOHC (12.44 min), **10**: CHB (12.56 min) and **11**: BP (12.86 min). Further peaks were detected; however, database comparisons were not unambiguous.

GC-MS

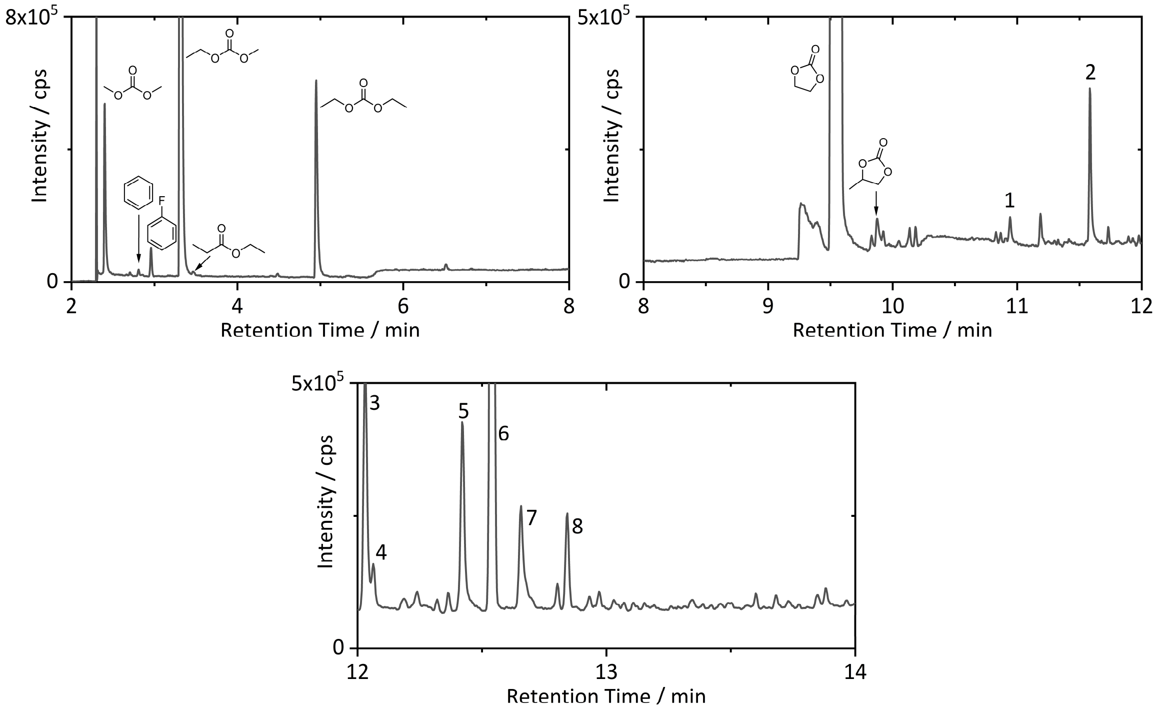

Figure S9: GC-MS chromatograms with liquid injection obtained from shredded LIBs after DCM extraction. For illustrative reasons the chromatogram is divided in 3 sections Species were identified based on NIST11 database comparisons and molecular formulas are depicted. Further species were identified as follows: **1**: Methylbenzaldehyde (10.94 min), **2**: DMDOHC (11.58 min), **3**: EMDOHC (12.03 min), **4**: Di-tert-butylbenzene (12.06 min), **5**: DEDOHC (12.42 min), **6**: CHB (12.54 min) **7**:ADN (12.66 min) and **8**: BP (12.84 min). Exact isomeric constitution of benzylic species was not evaluated. Further peaks were detected; however, database comparisons were inconclusive.

GC-HRMS

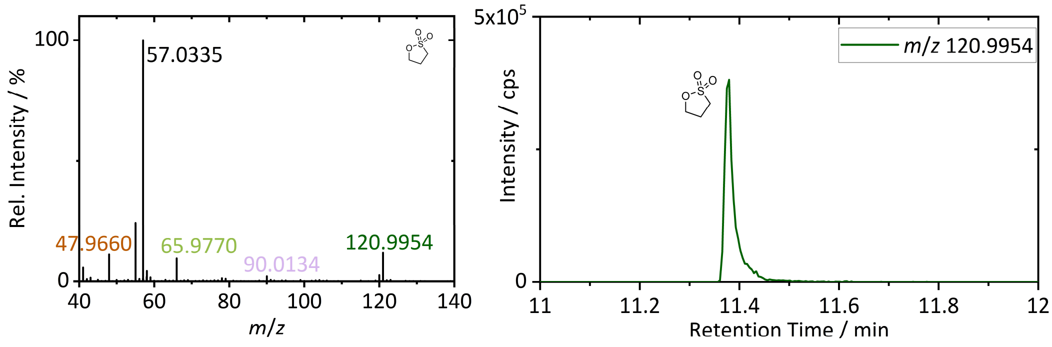

Figure S10: GC-HRMS mass spectrum of PS (I) obtained from measurement of the commercially available substance. Color code of sulfur containing marker fragment ions (*m/z* 47.9660 ($O^{32}S$), *m/z* 65.9770 ($H_2O_2{}^{32}S$), *m/z* 90.0134 ($C_3H_6O^{32}S$), *m/z* 120.9954 ($C_3H_5O_3{}^{32}S$)) is applied according to Figure 5. Further, the EIC (r.) with *m/z* 120.9954 form the GC-HRMS chromatogram from the standard substance was used for precise retention time determination (11.38 min).

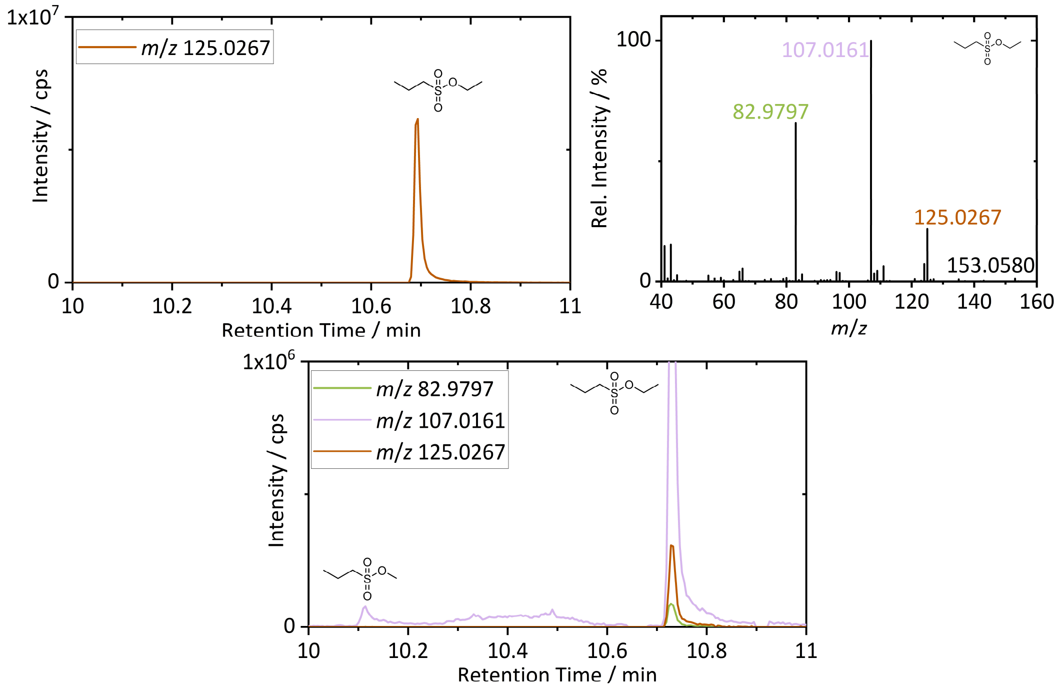

Figure S11: Identification of EPS by means of GC-HRMS. EPS was synthesized by stirring a mixture of 1-propanesulfonyl chloride (200 µL) and ethanol (2 mL). The reaction mixture was further diluted (1/100 *v*/*v*) and analyzed *via* GC-HRMS to obtain fragmentation behavior of EPS (top, l.) and the retention time (top, r.) on the GC-HRMS system. Especially, the EI fragmentation pattern differed compared to the GC-SQ-MS system (Figure S7). Sulfur containing marker fragment ions are marked in the obtained mass spectrum (top, right) and were applied for identification in the DCM extract (bottom).

IC-IT-TOF-MS

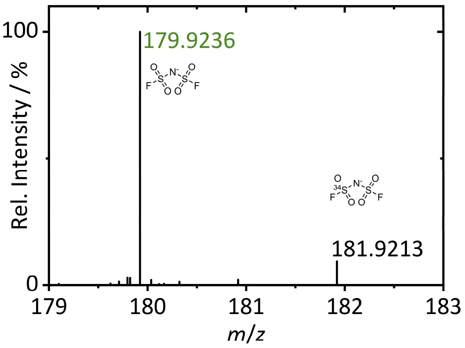

Figure S12: Background subtracted IC-IT-TOF mass spectrum of the peak at 25.6 min obtained *via* electrospray ionization in negative mode. Relative intensity of the ^{34}S containing ion, measured with *m*/*z* 181.9213, proved two sulfur atoms in the molecule. The M^- ion with measured *m*/*z* 179.9236 was chosen for chromatographic identification in Figure 8.

RPLC-IT-TOF-MS

Table S1: Identified oligo phosphates based on the target list introduced by *Henschel et al.*[3].

RT<br>min	formed<br>Adduct	*m*/*z*<br>meas.	*m*/*z*<br>calc.	Dev.<br>Δppm	Molecular formula	Group
5.3	$[M+Li]^+$	285.0475	285.0476	-0.35	C6H16O8P2	DiP
5.7	$[M+H]^+$	291.0376	291.0394	-6.18	C7H16O8P2	cycDiP
6.2	$[M+Li]^+$	299.0621	299.0632	-3.68	C7H18O8P2	DiP
7.1	$[M+H]^+$	307.0705	307.0706	-0.33	C8H20O8P2	DiP
8	$[M+H]^+$	321.0850	321.0862	-3.74	C9H22O8P2	DiP
8.7	$[M+H]^+$	335.1009	335.1018	-2.69	C10H24O8P2	DiP
6.8	$[M+Li]^+$	437.0707	437.0712	-1.14	C10H25O12P3	TriP
7.5	$[M+Li]^+$	451.0867	451.0868	-0.22	C11H27O12P3	TriP
8.1	$[M+Li]^+$	465.1032	465.1024	1.72	C12H29O12P3	TriP
8.7	$[M+Li]^+$	479.1201	479.1180	4.38	C13H31O12P3	TriP
9.3	$[M+Li]^+$	493.1341	493.1312	5.88	C14H33O12P3	TriP

Table S2: Identified phosphate carbonates based on the target list introduced by *Henschel et al.*[3].

RT<br>min	formed<br>Adduct	*m*/*z*<br>meas.	*m*/*z*<br>calc.	Dev.<br>Δppm	Molecular formula	Group
6.8/6.9	$[M+Li]^+$	249.0670	249.0711	-16.46	C7H15O7P1	1P+1C
7.8/8.0	$[M+Li]^+$	263.0874	263.0867	2.66	C8H17O7P1	1P+1C
9	$[M+Li]^+$	277.1023	277.1023	0.00	C9H19O7P1	1P+1C
8.3	$[M+Li]^+$	337.0875	337.0870	1.48	C10H19O10P1	1P+2C
9.2/9.3	$[M+Li]^+$	351.1033	351.1026	1.99	C11H21O10P1	1P+2C
10.0/10.2	$[M+Li]^+$	365.1190	365.1182	2.19	C12H23O10P1	1P+2C
7.3	$[M+H]^+$	381.0668	381.0709	-10.76	C10H22O11P2	2P+1C
8.1/8.3	$[M+H]^+$	395.0876	395.0865	2.78	C11H24O11P2	2P+1C
8.8/9.0	$[M+H]^+$	409.1027	409.1021	1.47	C12H26O11P2	2P+1C
9.5/9.7	$[M+H]^+$	423.1177	423.1178	-0.24	C13H28O11P2	2P+1C
10.1/10.2	$[M+Li]^+$	439.1203	439.1185	4.10	C14H25O13P1	1P+3C
8.3/8.4	$[M+Li]^+$	539.1042	539.1026	2.97	C14H31O15P3	3P+1C
8.9/9.0	$[M+Li]^+$	553.1190	553.1183	1.27	C15H33O15P3	3P+1C
9.4/9.5	$[M+Li]^+$	567.1335	567.1339	-0.71	C16H35O15P3	3P+1C
10.1	$[M+Li]^+$	581.1493	581.1495	-0.34	C17H37O15P3	3P+1C
10.7	$[M+Li]^+$	591.1422	591.1421	0.17	C18H34O17P2	2P+3C
11.4	$[M+Li]^+$	605.1577	605.1577	0.00	C19H36O17P2	2P+3C

Table S3; Identified cyclic carbonates based on the target list introduced by *Henschel et al.*[3].

RT<br>min	formed<br>Adduct	*m*/*z*<br>meas.	*m*/*z*<br>calc.	Dev.<br>Δppm	Molecular formula	Group
6.8/6.9	$[M+NH_4]^+$	326.1467	326.1445	-16.46	C7H15O7P1	cycC
11.4	$[M+NH_4]^+$	414.1221	414.1241	0.00	C19H36O17P2	cycC

Table S4: Identified ether carbonates based on the target list introduced by *Henschel et al.*[3].

RT min	formed Adduct	*m/z* meas.	*m/z* calc.	Dev. Δppm	Molecular formula	Group
4.4	$[M+H]^+$	209.1360	209.1384	-11.48	C9H20O5	X0Y4R1bR2a
9.7	$[M+H]^+$	251.1126	251.1125	0.40	C10H18O7	X2Y2R1cR2c
6.7	$[M+H]^+$	251.1846	251.1853	-2.79	C12H26O5	X0Y4R1cR2c
8	$[M+H]^+$	267.0689	267.0711	-8.24	C9H14O9	X3Y2R1bR2b
9.4	$[M+NH_4]^+$	298.1123	298.1133	-3.35	C10H16O9	X3Y2R1b/cR2b/c
10.7	$[M+H]^+$	295.1015	295.1024	-3.05	C11H18O9	X3Y2R1cR2c
10	$[M+H]^+$	295.1367	295.1387	-6.78	C12H22O8	X2Y3R1cR2c
7.1	$[M+H]^+$	295.2122	295.2115	2.37	C14H30O6	X0Y5R1cR2c
9	$[M+NH_4]^+$	342.1755	342.1757	-0.58	C13H24O9	X2Y4R1b/cR2b/c
10.5	$[M+NH_4]^+$	386.1280	386.1292	-3.11	C13H20O12	X4Y3R1b/cR2b/c
9.3	$[M+NH_4]^+$	386.1999	386.2018	-4.92	C15H28O10	X2Y5R1b/cR2b/c
11.5	$[M+NH_4]^+$	400.1444	400.1448	-1.00	C14H22O12	X4Y3R1cR2c
10.4	$[M+NH_4]^+$	400.2189	400.2174	3.75	C16H30O10	X2y5R1cR2c
7.8	$[M+NH_4]^+$	400.2908	400.2900	2.00	C18H38O8	X0Y7R1cR2c
6.7	$[M+NH_4]^+$	416.2857	416.2854	0.72	C18H38O9	X0Y8R1bR2b
9.2	$[M+NH_4]^+$	430.2291	430.2279	2.79	C17H32O11	X2Y6R1b/cR2b/c
7.3	$[M+NH_4]^+$	430.3015	430.3005	2.32	C19H40O9	X0Y8R1b/cR2b/c
8	$[M+NH_4]^+$	444.3156	444.3161	-1.13	C20H42O9	X0Y8R1cR2c
7	$[M+NH_4]^+$	460.3164	460.3116	10.43	C20H42O10	X0Y9R1bR2b
11.8	$[M+NH_4]^+$	474.1832	474.1814	3.80	C17H28O14	X4Y5R1b/cR2b/c
7.6	$[M+NH_4]^+$	474.3252	474.3266	-2.95	C21H44O10	X0Y9R1b/cR2b/c
10.6	$[M+NH_4]^+$	488.2335	488.2333	0.41	C19H34O13	X3Y6R1cR2c
8.3	$[M+NH_4]^+$	488.3417	488.3422	-1.02	C22H46O10	X0Y7R1cR2c
8.1	$[M+NH_4]^+$	504.1535	504.1559	-4.76	C17H26O16	X5Y5R1bR2b
7.2	$[M+NH_4]^+$	504.3409	504.3378	6.15	C22H46O11	X0Y10R1bR2b
8.7	$[M+NH_4]^+$	518.1662	518.1712	-9.65	C18H28O16	X5Y5R1b/cR2b/c
7.9	$[M+NH_4]^+$	518.3550	518.3527	4.44	C23H48O11	X0Y10R1b/cR2b/c
9.3	$[M+NH_4]^+$	532.1858	532.1868	-1.88	C19H30O16	X5Y5R1cR2c
8.4	$[M+NH_4]^+$	532.3642	532.3683	-7.70	C24H50O11	X0Y10R1cR2c
7.5	$[M+NH_4]^+$	548.3640	548.3641	-0.18	C24H50O12	X0Y11R1bR2b
11.6	$[M+NH_4]^+$	562.1878	562.1973	-16.90	C20H32O17	X5Y6R1b/cR2b/c
8.1	$[M+NH_4]^+$	562.3803	562.3788	2.67	C25H52O12	X0Y11R1b/cR2b/c
11.4	$[M+NH_4]^+$	576.2466	576.2492	-4.51	C22H38O16	X4Y7R1cR2c
8.6	$[M+NH_4]^+$	576.3951	576.3944	1.21	C26H54O12	X0Y11R1cR2c
7.7	$[M+NH_4]^+$	592.3893	592.3903	-1.69	C26H54O13	X0Y12R1bR2a
9.6	$[M+NH_4]^+$	606.1838	606.1871	-5.44	C21H32O19	X6Y6R1b/cR2b/c
8.2	$[M+NH_4]^+$	606.4063	606.4049	2.31	C27H56O13	X0Y12R1b/cR2b/c
10.1	$[M+NH_4]^+$	620.1929	620.2027	-15.80	C22H34O19	X6Y6R1cR2c
8.8	$[M+NH_4]^+$	620.4200	620.4205	-0.81	C28H58O13	X0Y12R1cR2c

References

[1] Y. P. Stenzel, M. Börner, Y. Preibisch, M. Winter, S. Nowak, *J. Power Sources* **2019**, *433*, 226709.

[2] S. Tsuge, H. Ohtani, C. Watanabe, *Pyrolysis-GC/MS Data Book of Synthetic Polymers: Pyrograms, Thermograms and MS of Pyrolyzates*, Elsevier, **2011**.

[3] J. Henschel, C. Peschel, F. Günter, G. Reinhart, M. Winter, S. Nowak, *Chem. Mater.* **2019**, *31*, 9977–9983.
